# Spontaneous generation of a novel foetal human retinal pigment epithelium (RPE) cell line available for investigation on phagocytosis and morphogenesis

**DOI:** 10.1111/cpr.12386

**Published:** 2017-09-18

**Authors:** Zhihua Shao, Haiyun Wang, Xuejian Zhou, Baosen Guo, Xuehu Gao, Zengrong Xiao, Meng Liu, Jihong Sha, Chunlian Jiang, Yuping Luo, Zhixue Liu, Siguang Li

**Affiliations:** ^1^ Stem Cell Translational Research Center Tongji Hospital Tongji University School of Medicine Shanghai China; ^2^ Shanghai First Maternity and Infant Health Hospital Tongji University School of Medicine Shanghai China; ^3^ Department of Regenerative Medicine Tongji University School of Medicine Shanghai China; ^4^ College of Life Sciences Nanchang University Nanchang China; ^5^ School of Life Sciences and Technology Tongji University Shanghai China

## Abstract

**Objectives:**

Primary retinal pigment epithelium (RPE) cells have a limited capacity to re‐establish epithelial morphology and to maintain native RPE function in vitro, and all commercially available RPE cell lines have drawbacks of morphology or function*;* therefore, the establishment of new RPE cell lines with typical characteristics of RPE would be helpful in further understanding of their physiological and pathological mechanisms. Here, we firstly report a new spontaneously generated RPE line, fhRPE‐13A, from a 13‐week aborted foetus. We aimed to investigate its availability as a RPE model.

**Materials and methods:**

RNA‐seq data were mapped to the human genome assembly hg19. Global transcriptional data were analysed by Weighted Gene Co‐expression Network Analysis (WGCNA) and differentially expressed genes (DEGs). The morphology and molecular characteristics were examined by immunofluorescence, transmission electron micrographs, PCR and western blot. Photoreceptor outer segments (POS) phagocytosis assay and transepithelial resistance measurement (TER) were performed to assess phagocytic activity and barrier function, respectively.

**Results:**

The fhRPE‐13A cells showed typical polygonal morphology and normal biological processes of RPE. Meanwhile they were capable of POS phagocytosis in vitro, and the expression level of TYR and TYRP1 were significantly higher than that in ARPE‐19 cells.

**Conclusions:**

The foetal human RPE line fhRPE‐13A is a valuable system for researching phagocytosis and morphogenesis of RPE in vitro.

## 1 INTRODUCTION

The retinal pigment epithelium (RPE) is a pigmented monolayer epithelium with notably polygonal morphology, which locates between the neural retina and the choroid. RPE plays a critical role in nourishing, supporting, protecting neural retina,^1^ immunoregulation and proinflammatory reaction.^2^, ^3^ The loss or dysfunction of RPE are often causes of visual problem, such as age‐related macular degeneration (AMD)^4^ and retinitis pigmentaosa (RP).^5^ Therefore, it is significant to fully understand and demonstrate the physiology and pathophysiology mechanisms of RPE.

Cells in culture are important models for investigating the mechanisms of RPE in vitro. Primary cultured foetal or adult RPE cells retain typical appearance and markers of RPE,^6^, ^7^ but they appear to be lack of their polygonal morphology and become motile fibroblast‐like cells if they are cultured at a low density or repeated passage.^8^, ^9^ The proliferation of primary RPE cells is limited to about 5 to 6 passages^10^; and they tend to adopt mesenchymal fates regardless of RPE sources.^7^ This transition from an epithelial to a mesenchymal‐like phenotype accompanies the alteration of cellular functions, such as cell‐cell junctions, cytoskeletal rearrangement as well as expression of genes associated with epithelial‐mesenchymal transition.^8^, ^9^, ^11^ From this point, RPE cell lines with polygonal morphology and normal function are indispensable resource in the field of RPE research.

At present, human RPE cell lines, such as h1RPE‐7, h1RPE‐116, ARPE‐19 and D407 have become available.^12^, ^13^, ^14^ But, only ARPE‐19 is a popular used cell line and has been shown to have morphological and functional properties of native RPE, including the ability to phagocytosis photoreceptor outer segments^15^ and to secrete endogenous growth factor.^6^ However, ARPE‐19 lacks some important properties of the native RPE. For instance, it is absent from pigmentation and RPE markers, such as RPE65 (RPE‐specific protein 65 kDa), has low TER as well.^6^ These defects significantly limit the application of the ARPE‐19 cells.

In the present study, we firstly reported a novel foetal human RPE cell line, fhRPE‐13A, which was generated spontaneously from a primary RPE culture. We described its characters, and observed that the fhRPE‐13A had normal morphology and gene expression of RPE. Furthermore, we performed global transcriptome profiling analysis of the fhRPE‐13A at different stages. Gene ontology analysis revealed that the normal biological processes of RPE were highly correlated with fhRPE‐13A cells, enriched with terms of ion transport, pigmentation, phagocytosis, vitamin A and retinoid metabolism. In addition, the availabilities of fhRPE‐13A were evaluated by performing a comparative analysis of transcriptomes and experimental analysis. All results suggested that fhRPE‐13A cells possess normal epithelial morphology and the activity of phagocytosis. Our study provided a valuable culture system for studying on physiological function of RPE, such as phagocytosis and morphogenesis.

## 2 MATERIALS AND METHODS

### 2.1 Native foetal tissue and RPE Primary culture

This research followed the tenets of Tongji University Institutional Review Board. Human foetal eyes (gestation, 10‐13 weeks) were obtained from Shanghai First Maternity and Infant Health Hospital. Human foetal native RPE (fhRPE) was isolated following previous protocol.^16^ The isolated fhRPE was cut into 1‐2 mm diameter sheets, then seeded in 24‐well plates (BD Bioscience, San Jose, CA) and cultivated with Dulbecco's modified Eagle's medium/Hams F‐12 (DMEM/F‐12; Life technologies, Calrsbad, CA) containing 1% Penicillin‐Streptomycin (Gibco, Rockville, MD) and 15%‐20% foetal bovine serum (FBS, Gibco) at 37°C in 5% CO_2_. When certain amount of individual cells with pigment migrated from attached sheets, the 15%‐20% FBS was replaced to 10% FBS.

### 2.2 Cell subcultures

Primary fhRPE cells cultured to day 28 were separated into 1‐2 mm diameter sheets using a glass needle under The EVOS Cell Imaging System (Life Technologies), then passaged in a 24‐well plate at 1:2 ratios. After passage 10, the RPE cells were digested with 0.05% Trypsin‐EDTA (Gibco) no more than 2 minutes. Two RPE clones fhRPE‐13A and fhRPE‐13B, derived from a 13‐week donor, were generated, and cultivated in DMEM/F12 with 10% FBS. Originally, both cells were passaged in a 24‐well plate at 1:2 ratios, then cells were cultured at a density of 2.5‐5 × 10^4^ cells/cm^2^ during extended culture in a 6‐well plate or 10 cm culture dish. Since the phenotype of RPE is dependent on culture medium, we also cultivated fhRPE‐13A cells in Dulbecco's Modified Eagle's Medium with high glucose and pyruvate (H‐DMEM, GE Healthcare Life Sciences, South Logan, UT) with 10% FBS. ARPE‐19 and passaged adult human RPE (ahRPE) were gifts kindly given by other lab of Tongji University School of Medicine. Both cells were routinely passaged with 0.05% Trypsin‐EDTA and cultivated in DMEM/F12 with 10% FBS at a density of 2.5‐5 × 10^4^ cells/cm^2^. For longer term culture, post‐confluent cultures were maintained in media at a final concentration of 1% FBS in a humidified incubator, kept at 37°C in 5% CO_2_. The medium was half changed every 2 days.

### 2.3 RNA‐Seq library construction, sequencing and mapping

The samples in this study including fhRPE‐13A, fhRPE‐13B, ARPE‐19 and passaged ahRPE were cultivated in DMEM/F12 with 10% FBS. The fhRPE‐13A cells (Passage15, P15; Passage27, P27; Passage48, P48), fhRPE‐13B cells and ahRPE cells were separately collected on days 2, 3, 4 and 5. The ARPE‐19 cells were collected on day 5. Total RNA was extracted using TRIzol^®^ Regeant (Invitrogen, Carlsbad, CA), whereafter, RNA‐seq libraries were constructed by the Annoroad Gene Technology (Annoroad, Beijing, China). Libraries were sequenced on the Illumina HiSeq2000 and single‐end reads of 50 bp were obtained. All reads were mapped to the human genome assembly hg19 with Tophat (version 2.1.0) as published protocol.^17^ The mapping rates reached to >90% for each sample. The RNA‐seq data in this paper have been deposited in NCBI's Gene Expression Omnibus (GEO) and are accessible through GEO Series accession number GSE102875.

### 2.4 Data analysis

A signed weighted correlation network was constructed using Weighted Gene Co‐expression Network Analysis (WGCNA) R package.^18^ To minimize the noise and spurious associations, the adjacency matrix was established with the power β = 12. Then, the adjacency matrix was transformed to topological overlap matrix (TOM) which is a biologically meaningful measure of network interconnectedness. Modules were produced by grouping together highly similar co‐expression relationships on the topological overlap. Subsequently, modules with highly correlation (correlation > 0.85) were merged together. The differentially expressed genes were identified by DEGseq R package as previously described.^19^ The Database for Annotation, Visualization and Integrated Discovery (DAVID, http://david.abcc.ncifcrf.gov/) 20 was used to perform functional enrichment analysis on the interest genes, and the significantly enriched functions using a cut‐off criterion of *P *< .05.

### 2.5 End‐point RT‐PCR and Quantitative PCR

Total RNA was extracted using TRIzol^®^ Regeant (Invitrogen). RNase free DNase kit (TaKaRa, Kusatsu, Shiga, Japan) was used to remove contaminating genomic DNA. cDNA was synthesized using 5XPrimeScript^™^ RT Master Mix (TaKaRa). End‐point RT‐PCR was carried out using 0.2‐1.0 μg of total RNA in the PCR reaction systems (Life Technologies). The PCR products were visualized on 1%‐2% agarose gels. Quantitative PCR was performed using 2XSYBR Premix EX Taq^™^ II kit (Takara) according to the standard protocol on Quantitative PCR System (ABI, 7900; Applied Biosystems, Foster City, CA). Each sample was run in triplicate and non‐transcriptase reaction was used as control. Relative changes levels of each gene were calculated with ΔΔCt method. The primer sequences are shown in Table S1.

### 2.6 Immunofluorescence

Coverslips (Fisher Scientific) were coated with poly‐ornithine (1:1000 in water, Sigma‐Aldrich, St. Louis, MO) at 37°C for 4 hours and washed three times with water, then they were coated with laminin (1:200 in PBS, Sigma–Aldrich) at room temperature at least 4 hours. Cells were seeded at a density of 2.5‐5 × 10^4^ cells/cm^2^ and kept at 37°C in 5% CO_2_. As the expression of RPE specific protein is related to the culture time, cell climbing slices were fixed for 20 minutes in 4% paraformaldehyde (Sigma–Aldrich) when cells were confluent about 5 to 6 days. Slices were penetrated with 0.1% Triton‐×100 (Sigma–Aldrich) for 10 minutes, then incubated with blocking solution (3% goat or donkey serum in PBS) at room temperature for 1 hour. Primary antibodies were incubated overnight at 4°C. After washed with PBS, slices were incubated for 1 hour with secondary antibodies in blocking solution at room temperature. Nuclei were counterstained with DAPI (Roche, Mannheim, Germany). Images were analysed on Fluorescence Microscope (Nikon, Tokyo, Japan) or Confocal Laser Scanning Microscope (Leica, Wetzlar, Germany). Primary and secondary antibodies are shown in Table S2.

### 2.7 Western blot analysis

Monolayers of fhRPE‐13A and ARPE‐19 cultivated in DMEM/F12 or H‐DMEM with 10% FBS at day 14 or day 5 were harvested and suspended in a RIPA Lysis Buffer (Beyotime, Shanghai, China) with 1 mmol L^−1^ PMSF (Beyotime). Proteins were separated on the 8% or 7% SDS‐polyacrylamide gel, then transferred to a PVDF membrane (Millipore, Bedford, MA). Subsequently, the membrane was blocked for 1 hour in 5% skim milk at room temperature and probed with primary antibodies overnight in 0.5% skim milk at 4°C, followed by the incubation of horseradish‐peroxidase‐conjugated secondary antibodies for 1 hour in 5% blocking buffer at room temperature. Protein bands were detected using a ECL chemiluminescence kit (Thermo Scientific, MA) with Image Quant LAS4000mini (GE Healthcare Life Sciences, UT) system. Primary and secondary antibodies are shown in Table S2.

### 2.8 Transmission electron microscopy

ARPE‐19 and fhRPE‐13A were cultured at a density of 2.5 × 10^4^ cells/cm^2^ on a Transwell culture system (24‐mm inserts, Corning, Lowell, MA) in DMEM/F12 with 10% FBS. Before seeding, the transwells were coated with human extracellular matrigel (7 uL in 200 uL serum free DMEM/F12 per well, BD Biosciences). The medium was half changed every 2 days. Cell monolayers cultured for 14 days and native fhRPE were fixed in 3% glutaraldehyde‐buffered solution (Ted Pella, Redding, CA) at 4°C for overnight. A routine ultra‐thin sections sample preparation protocol was used. Specimens were cut (80 nm) and stained, then examined on Transmission Electron Microscopy (JEOL, Tokyo, Japan).

### 2.9 G‐banding karyotype

Confluent cells treated with 0.2 ug/ml colchicine(Sigma–Aldrich) for 2 hours were digested with 0.05% Trypsin‐EDTA (Gibco), then suspended in 0.075 mol L^−1^ potassium chloride for 25 minutes at 37°C. After fixation with methyl alcohol and glacial acetic acid in 3:1 ratio for 30 minutes, the karyotypes were analysed in the Da An Health Testing Center Shanghai, China.

### 2.10 Photoreceptor outer segments phagocytosis assay

Fresh porcine eyes were obtained from a local slaughterhouse. Photoreceptor Outer Segments (POS) were isolated and purified as a paper reported.^21^ POS was diluted to 2 × 10^7^ POS/mL in PBS and was labelled using 200 uL of the 10 mmol L^−1^ NHS‐LC‐LC‐Biotin (Thermo Scientific, MA) reagent solution per mL for 30 minutes at room temperature. Labelled POS were washed to quench and remove excess biotin reagent and byproducts with 100 mmol L^−1^ glycine (Sigma–Aldrich) in PBS. Finally, labelled POS were suspended in 4% FBS containing H‐DMEM at a density of 1 × 10^7^ POS/mL. fhRPE‐13A cells and HEK 293FT (negative control) were cultivated onto coverslips (Fisher Scientific, NH) coated with poly‐ornithine (1:1000 in water, Sigma–Aldrich) and laminin (1:200 in PBS, Sigma–Aldrich) in a 24‐well plate with 10% FBS containing H‐DMEM. The day before adding labelled POS, culture medium was replaced with 4% FBS containing H‐DMEM. Labelled POS were added on confluent monolayer of fhRPE‐13A cells(day 5 to day 7) and HEK 293FT with 3 × 10^6^ POS per well and incubated at 37°C in 5% CO_2_ for 4 and 8 hours, respectively. To wash off the non‐specific adhesion, the cells were washed with PBS, then fixed with 4% paraformaldehyde (Sigma–Aldrich) for 20 minutes and blocked with 3% donkey serum at room temperature for 1 hour. To discriminate between surface binding and internalization, cells firstly were stained with opsin antibody no more than 1 hour at room temperature before penetration. After washing 3‐5 times, cells were penetrated and incubated with appropriate primary antibody, the steps were same with Immunofluorescence. Stained cells were examined with Confocal Laser Scanning Microscope (Leica). Primary and secondary antibodies are shown in Table S2. To calculate total POS, bound POS and internal POS per cell, total and surface‐bound POS and cell nuclei were counted at least 50 cells in each sample. And, one hundred cells were counted to calculate the numbers of phagocytic cell. Every sample was performed in triplicate.

### 2.11 Transepithelial resistance (TER)

Cells were plated in Transwell system (24‐mm inserts, Corning) coated with Matrigel (7 uL in 200 uL serum free DMEM/F12 per well, BD Biosciences) at a density of 2.5 × 10^4^cells/cm^2^ in DMEM/F12 with 10% FBS. The medium was changed to 1% FBS containing medium after first 48 hours and changed twice a week. The TER was monitored by measurement of an epithelial volt/ohm meter using an Electronic Volt–Ohmmeter (Millipore, Bedford, MA) as previously described.^16^

## 3 RESULTS

### 3.1 Two fhRPE‐13 lines with different morphology are spontaneously generated from a human foetal donor

**Figure 1:**
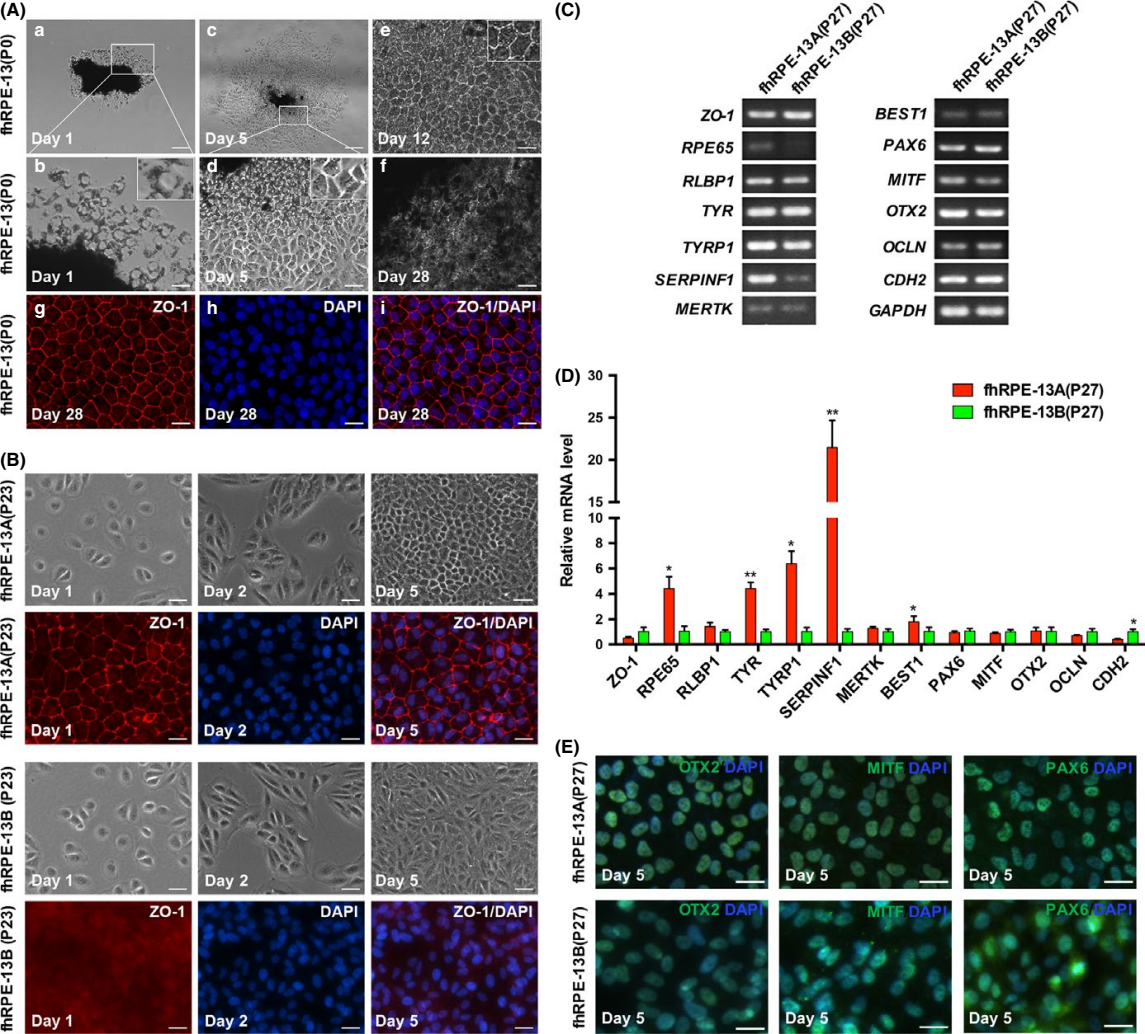
Generation of fhRPE‐13A and fhRPE‐13B lines from a fetal human donor. A, Primary culture of fhRPE‐13. Scale bars: 200 μm (c), 100 μm (a), 50 μm (d), 25 μm (b, e‐i). B, Distinct morphology of fhRPE cell lines (fhRPE‐13A and fhRPE‐13B) during subculture. Scale bars: 50 μm. C, RT‐PCR assay for detecting the expression of RPE genes in fhRPE‐13A and fhRPE‐13B. D, Quantitative RT‐PCR analysis of expression levels of RPE genes in fhRPE‐13A and fhRPE‐13B. The expression levels of genes in all lines are calculated and shown as mean ± SD. *P < .05, **P < .01. E, Immunofluorescence staining of fhRPE‐13A and fhRPE‐13B cells using antibodies against OTX2, MITF and PAX6. Scale bars, 50 μm

We mechanically separated the RPE monolayers into small sheets and cultured them in 24‐well plates. Within first 24 hour at primary culture, the foetal RPE sheets were attached like an island, and several individual cells with a large nucleus and heavy pigment migrated from the RPE sheets (Figure 1A, a and b). As the cells continued to divide, pigment density gradually decreased and the epithelial morphology was lost (Figure 1A, c and d). After the next few days, the cells obtained the pigment and rebuild polygonal shape (Figure 1A, e), and finally matured to be pigmented RPE (Figure 1A, f‐i).

As described in many papers, most primary RPE cells from foetal eyes hardly propagate after 5 to 6 passages.^10^ Surprisingly, two RPE cell clones, originally from a 13‐week foetal donor, named fhRPE‐13A and fhRPE‐13B, proliferated rapidly. Interestingly, like primary cultures of RPE, fhRPE‐13A exhibited polygonal epithelial morphology consisting mostly of hexagon (Figure 1B and Figure S1A) at days 5 to 6, even though they were repeatedly passaged (Figure S1B). However, the fhRPE‐13B cells exhibited atypically fibroblast‐like shape at day 5 (Figure 1B), and this de‐epithelial morphology maintained to higher passages when they were seeded at a same density of 2.5 × 10^4^ cells/cm^2^ with the fhRPE‐13A cells (Figure S1C). A comparison of RPE genes which have been verified experimentally to be expressed in foetal RPE,^6^, ^7^, ^16^, ^22^, ^23^, ^24^, ^25^ showed that *RPE65* (Retinoid Isomerohydrolase), *TYR* (Tyrosinase), *TYRP1* (Tyrosinase Related Protein 1), *SERPINF1* (Serpin Family F Member 1), *BEST1* (Bestrophin 1) were expressed significantly higher in fhRPE‐13A cells than in fhRPE‐13B cells (Figure 1C and D). Additionally, given that primary cultures of RPE always promote growth of choroidal fibroblast contaminants, we examined OTX2 (orthodenticle homolog 2), PAX6 (Paired Box6) and MITF (microphthalmia‐associated transcription factor) using immunofluorescence stain, which are necessary for RPE specification and drive expression of the functional proteins of RPE,^26^, ^27^ as well as lack for choroidal fibroblast. The results showed that both cells homogeneously expressed these transcription factor (Figure 1E). We also analysed the G‐banding karyotype, and found that both cell lines existed a diploid karyology (46, XY), but possessed abnormal chromosomal morphology (Figure S1D).

### 3.2 fhRPE‐13A cells display the normal biological processes of RPE

**Figure 2:**
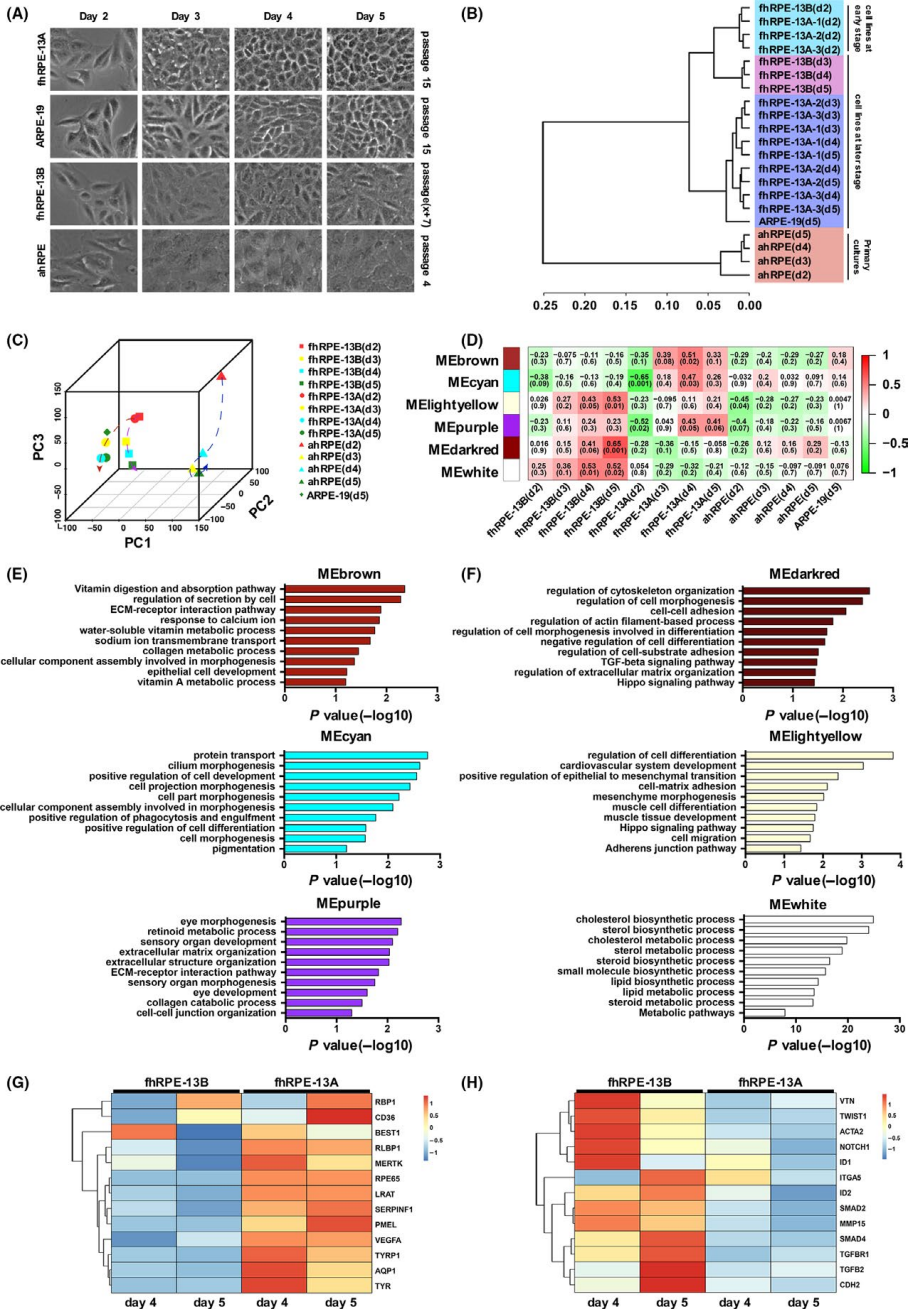
fhRPE‐13A cells have the normal biological processes of RPE. A, The morphology of fhRPE‐13A(P15), fhRPE‐13B(P15), ARPE‐19(PX+7) and ahRPE(P4) on day 2,3,4 and 5. B, Unsupervised hierarchical clustering of the transcriptomes of all samples. C, Principal component analysis (PCA) of four types of cells from different stages. Cells with same sample types are shown in same shape. Cells in same time point are shown in same color. Dash lines with an arrow indicate the culture time process of each cell line. D, Stage‐specific co‐expression gene modules identified by WGCNA and their correlation to the culture stage. The number of each square represents a correlation of modules and sample types, and *P*‐value of each correlation represents a correlation value. The color of each square is corresponding to the correlation: Positive correlation (Red); Negative correlation (Green); No correlation (White). E and F, Six significant GO categories identified in modules. MEbrown modue, MEcyan modue and MEpurple modue are related with fhRPE‐13A(E). MEdarkred modue, MElightyellow modue and MEwhite modue are related with fhRPE‐13B (F). The length of bars indicates −log_10_ *P* value. G and H, Expression patterns of general RPE markers(G) and epithelial‐mesenchymal transition markers(H) at different stages of the fhRPE‐13A and fhRPE‐13B lines. Colors represent gene expression levels: High expression (Red); Moderate expression (White); Low expression (Blue)

To obtain molecular insights into why RPE cell lines from the same donor have distinct morphology and expression patterns of RPE cell markers, we performed RNA‐seq. Here, we collected four different types of RPE cultures, including fhRPE‐13A, fhRPE‐13B, ARPE‐19 and primary adult human RPE (ahRPE). ARPE‐19, a well‐known human RPE cell line,^13^ can develop to polygonal RPE, served as normal control; the ahRPE at four passages, which lost the polygonal epithelial morphology (Figure 2A), served as abnormal control. The hierarchical clustering (HC)^28^ and Principal‐Component Analysis (PCA)^29^ are frequently used methods to cluster subgroups on the relationship and properties of each sample at different dimensions. HC and PCA analyses showed that these cell samples were divided into four groups depending on cell types and different culture time points (Figure 2B and C). The passaged primary ahRPE cells were accurately separated from fhRPE‐13 and ARPE cell line groups. In addition, fhRPE‐13A, fhRPE‐13B and ARPE‐19 cell lines were clustered together at early culture time (day 2), but categorized as two distinct groups (fhRPE‐13A and fhRPE‐13B) at later culture time (days 3, 4 and 5), which was consistent with their properties and the culture process.

To further ascertain the biological features of two novel cell lines, we conducted Weighted Gene Co‐expression Network Analysis (WGCNA)^18^ for detecting the gene co‐expressed modules of fhRPE‐13A and fhRPE‐13B cells along different time points. Obviously, we identified nine co‐expression gene modules (MEbrown, MEcyan, MEpurple, MEblue, MEmagenta, MEmidnightblue, MEdarkred, MElightyellow and MEwhite) with the highest correlation with fhRPE‐13A and fhRPE‐13B cell lines (Figure 2D and Figure S2A) based on time points and sample types. To determine the function of these gene modules, we performed gene ontology (GO) classification and enrichment analysis according to different cell types (Figure 2E and F and Figure S2B). In fhRPE‐13A groups, we found many terms related to core RPE function were enriched (MEbrown, MEcyan and MEpurple) (Figure 2E), including “vitamin A metabolic process”, “regulation of secretion by cell”, “sodium ion transmembrane transport”, “cilium morphogenesis”, “positive regulation of phagocytosis, engulfment”, “pigmentation”, “cell morphogenesis” and “retinoid metabolic process”. On the contrary, fhRPE‐13B groups (MEdarkred and MElightyellow, MEwhite), mainly enriched “positive regulation of epithelial to mesenchymal transition”, “mesenchyme morphogenesis, “muscle cell differentiation”, “muscle tissue development” and “cell migration”, and none of GO terms were associated with RPE function (Figure 2F), suggesting these cells may evolve toward a mesenchymal fate. In addition, the fhRPE‐13A cells highly expressed genes that are known to be important for RPE function (Figure 2G); some epithelial‐mesenchymal transition (EMT) related transcription factors^8^, ^30^ as well as up‐regulated expression genes during EMT were highly expressed in fhRPE‐13B cells (Figure 2H). Although EMT related genes, ITGA5 and ID1, also showed the tendency to increase in fhRPE‐13A, they finally decease when fhRPE‐13A cells completely differentiate at day 5. The difference of gene expressions further supported the idea that fhRPE‐13B cells were mesenchymal‐like RPE.

**Figure 3:**
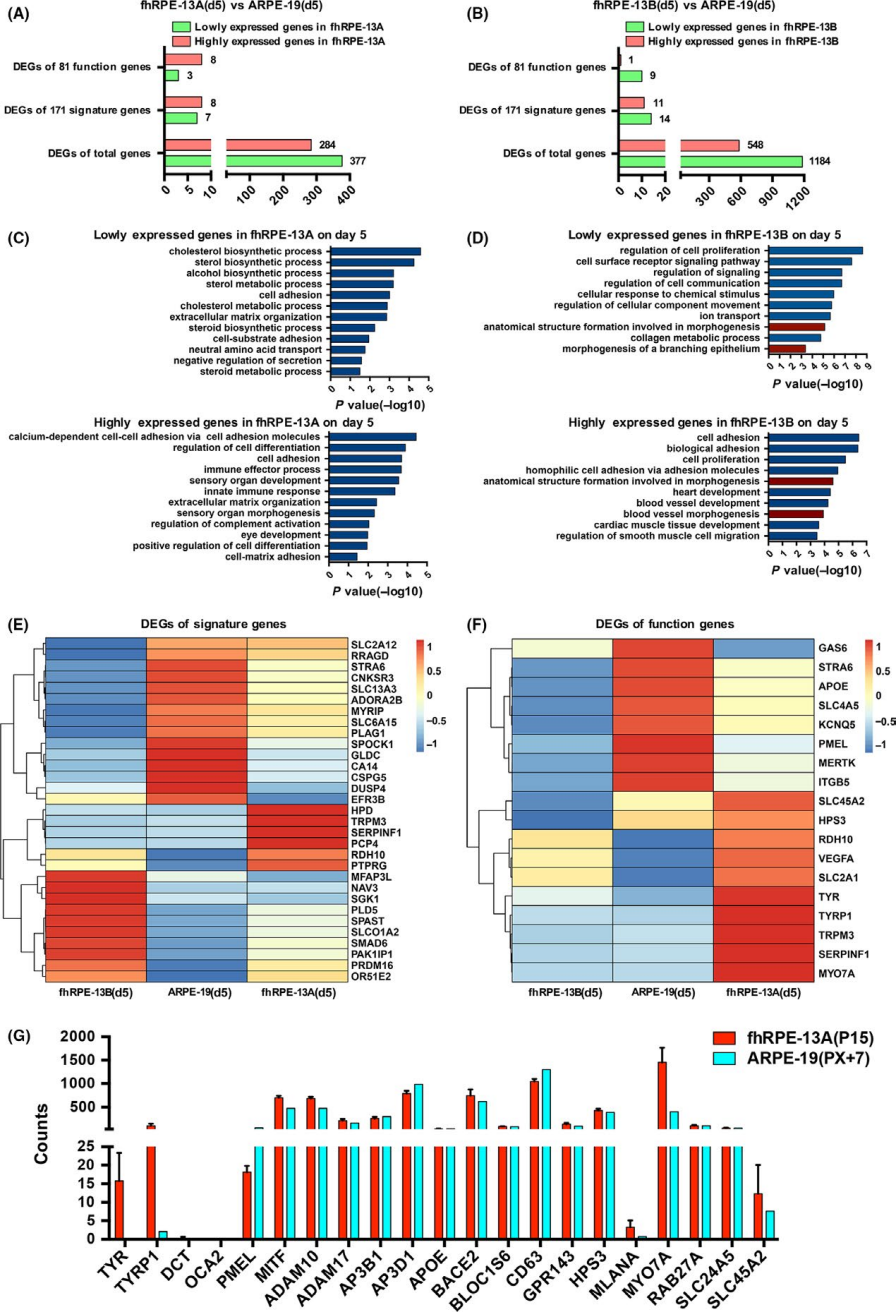
Gene expression profile analysis of DEGs between fhRPE‐13 cell lines and ARPE‐19 at day 5. A and B, Bar chart shows the number of differentially expressed genes (DEGs, FC ≥ 2 or FC ≤ 0.5, FDR < 0.05, FC = Fold Change) of total genes, signature genes and function genes between fhRPE‐13A (A) or fhRPE‐13B(B) and ARPE‐19, respectively. C and D, The Gene Ontology (GO) analys‐es of highly and lowly expressed DEGs in fhRPE‐13A (C) or fhRPE‐13B(D) cells on day 5. The significant GO categories identified in each of the 2 groups are shown. Red‐colored GO terms are associated with morphogenesis. The length of bars indicates −log_10_ *P* value of the Fisher's exact test. E and F, Heatmaps of DEGs about signature(E) or function genes (F) between both fhRPE‐13 lines and ARPE‐19 on day 5. Colors represent gene expression levels: High expression (Red); Moderate expression (White); Low expression (Blue). G, The expression levels of 21 pigmentation‐associated genes in fhRPE‐13A and ARPE‐19

To verify the result of WGCNA, we further compared the transcriptomes of fhRPE‐13A and fhRPE‐13B with ARPE‐19, respectively. A total of 661 differentially expressed genes (DEGs) were identified in fhRPE‐13A cells, of which 377 genes were lowly expressed and 284 genes were highly expressed (Figure 3A); a total of 1732 DEGs were found in fhRPE‐13B cells, of which 1184 genes were lowly expressed and 548 genes were highly expressed (Figure 3B). The numbers of DEGs were obviously enlarged in fhRPE‐13B cells, indicating a variation that deviated from the ARPE‐19 cells in the gene expression profiles. GO analysis on the DEGs was consist with the detection of WGCNA analysis, which appeared the fhRPE‐13B cells had mesenchymal cell fate and aberrant morphogenesis (Figure 3C and D).

### 3.3 fhRPE‐13A possess unique biological characteristics of RPE

RPE signature genes are a group of highly expressed genes in RPE determined by comparing the gene profiles of RPE with other tissues and cell types using stringent selection criteria.^31^, ^32^, ^33^ These genes consisted mostly of candidate genes for RPE disease, in which mutations lead to retinal disorders (eg, Age related macular degeneration, AMD). This set of genes was seemed as a validation on creation and used as a representative disease model. From this point, to find similarities and differences between fhRPE‐13 lines and ARPE‐19 on signature genes should be significant for their applications. To test this, we compared 171 “signature genes”^31^between fhRPE‐13 lines and ARPE‐19, and found 15 (8.77%) DEGs in fhRPE‐13A cells and 25 (14.6%) DEGs in fhRPE‐13B cells (Table S3, Figure 3A,B and E). According the paper,^32^ among these DEGs, *PTPRG, CSPG5* and *NAV3* are at loci associated with retinal disease; *MFAP3L, CSPG5* and *PAK1IP1* locate in the regions carrying SNP's significantly associated with AMD.

In addition, we analysed 81 functional genes expressed in RPE (Table S4), which are associated to pigmentation, transmembrane transport, barrier, secretion, retinoid cycle and phagocytosis of shed photoreceptor outer segments, and we separately found 11 (12.9%) and 10 (12.3%) DEGs in the fhRPE‐13A cells and the fhRPE‐13B cells compared with the ARPE‐19 cells (Figure 3A and B). In fhRPE‐13A cells, the majority of DEGs were highly expressed (Figure 3F), of particular note, two enzymes involved in melanogenesis, TYR and TYRP1, were at least 43‐fold higher expression in fhRPE‐13A than that in ARPE‐19 (Figure 3G). In fhRPE‐13B, undoubtedly most DEGs were decreased (Figure 3F). This seems that fhRPE‐13A cells possess the potential to be better to synthesize pigment than ARPE‐19 cells.

To more comprehensively and accurately evaluate the function of the fhRPE‐13A cells, we compared transcriptomes of fhRPE‐13A with primary fhRPE, which has been reported to most closely represent the healthy RPE in vivo, regardless of the mRNA expression profile or functions.^32^, ^34^ The RNA‐seq data of fhRPE cells at passage 0, which had been cultured for 4 weeks in vitro, were downloaded from NCBI Gene Expression Omnibus (GEO, GSE36695). 11 449 (63.9%) equally expressed genes, 3466 (19.4%) lowly and 2997 (16.7%) highly significant DEGs in fhRPE‐13A were identified (Figure S3A). The GO analysis of highly expressed genes in fhRPE‐13A revealed that the fhRPE‐13A cells are mitotically active, along the significantly enriched GO categories “cell cycle”, “cell division” and “DNA replication”, as well as active in “cytoskeleton organization”, “regulate signal transduction”, “junction assembly”, “cellular response to stress”, “cell‐cell adhesion” and “cell morphogenesis” (Figure S3B). To further details, we then focused on the 81 function genes and found that most phagocytosis‐associated genes were highly or equally expressed when compared with primary fhRPE (Figure S3C and D). These results suggest that fhRPE‐13A cells seems to be normal in activity of phagocytosis.

### 3.4 Characterization of fhRPE‐13A

**Figure 4:**
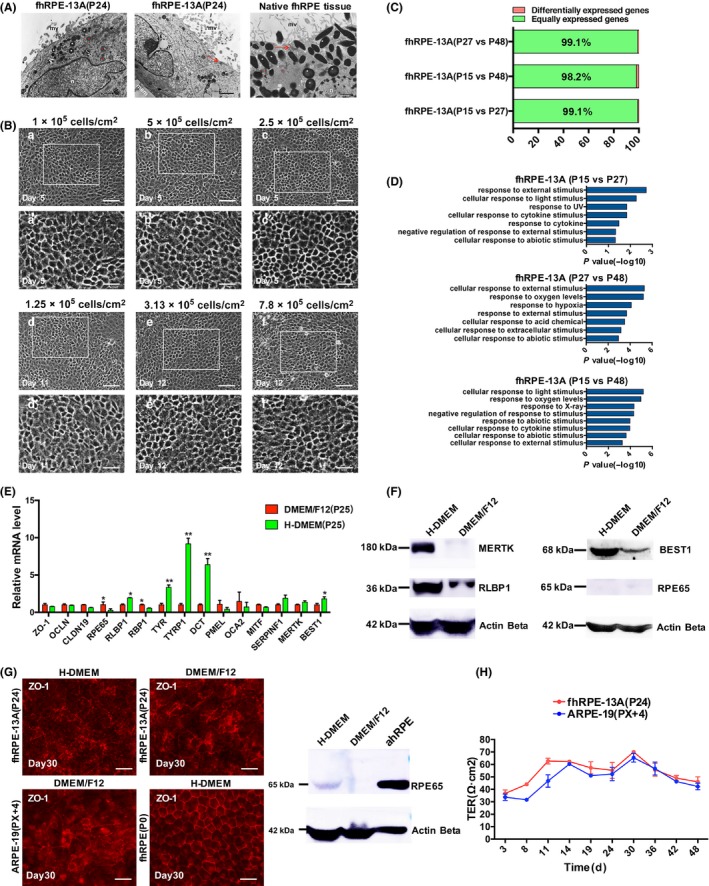
Characterization of fhRPE‐13A cultured in vitro. A, Transmission Electron Microscopy graphs of submicroscopic structure of fhRPE‐13A and native human fetal RPE. White asterisk, pigment granules; red asterisk, mitochondrion; red arrows, tight‐junction complexes. mv, microvilli; n, nucleus; mem, polyester membrane. Scale bars: 1 μm (a, b); 0.5 μm (c). B, Morphology of fhRPE‐13A cells seeded at various densities. Scale bars, 200 μm (a‐f);50 μm (a’‐f’). C, Bar chart shows the number of equally expressed genes and differentially expressed genes (DEGs, FC ≥ 2 or FC ≤ 0.5, FDR < 0.05, FC = Fold Change) between various passages. D, The Gene Ontology (GO) analysis of DEGs in various passages of fhRPE‐13A cells on day 5. The significant GO categories identified in each of the 2 groups are shown. The length of bars indicates −log_10_ *P* value of the Fisher's exact test. E, Quantitative RT‐PCR analysis of expression levels of drastically expressed RPE functional genes in H‐DMEM and DMEM/F12. The expression levels of genes in all samples are calculated and shown as mean ± SD. *P < .05, **P < .01. F, Western blot detection of MERTK, RLBP1, BEST1 and RPE65 proteins in fhRPE‐13A (P34) cultured in H‐DMEM or DMEM/F12. Total protein extract (50‐100 μg) of each sample at day 5 was used. G, Immunofluorescence staining of ZO‐1 in fhRPE‐13A(P24), ARPE‐19 (PX+4) and primary fetal RPE cells and western blot analysis of RPE65 protein in fhRPE‐13A cultured in H‐DMEM or DMEM/F12 at day 30. Adult RPE tissue(ahRPE) was used as control. Total protein extract (50‐100 μg) of each sample was used. Scale bars, 50 μm. H, The transepithelial resistance of fhRPE‐13A or ARPE‐19

The morphology of the fhRPE‐13A cells was evaluated with transmission electron microscopy. Similar to native fhRPE, fhRPE‐13A presented the apical side of mitochondrion, pigment granules, microvilli, tight‐junction complexes, and exhibited basally located nuclei, but the pigment accumulation was limited compared to native fhRPE (Figure 4A).

As primary RPE cells seeded at less than 5% density will dedifferentiate,^34^ this prompt us to question whether fhRPE‐13A will not differentiate into a functional monolayer at lower seeding density. To test this assumption, we seeded fhRPE‐13A cells in 24‐well plate from low to high density. Cells seeded at different densities could re‐differentiate into proper cobblestone morphology, nothing but cells seeded at 1.25‐7.8 × 10^4^/cm^2^ required more time for differentiation (Figure 4B). We chose density 2.5‐5 × 10^4^/cm^2^ in this study, at which less time and cells were required to perform cell culture.

The stability of gene expression profiles of fhRPE‐13A cells at different passages (Passage15, P15; Passage27, P27; Passage48, P48) were assessed with DEGs. No more than 2% DEGs were found among different passages (Figure 4C). The GO enrichment analysis indicated that terms related to response to external stimulus were enriched (Figure 4D), mainly including “response to light, UV or X‐ray”, “response to cytokine”, “response to abiotic stimulus”, “response to oxygen level and hypoxia”, and none of GO terms associated with RPE function were enriched.

Additionally, some molecules expressed in RPE cells are sensitive to culture medium, especially, RPE65, an essential isomerohydrolase, which was prompted when ARPE‐19 was cultured with H‐DMEM/pyruvate maintained medium for 15 weeks.^35^ Therefore, we compared the genes and proteins associated with RPE function of fhRPE‐13A in DMEM/F12 and H‐DMEM when cells were cultured for 5 days. Notably, the genes required for pigmentation including *TYR, TYRP1* and *DCT*; for visual cycle including *RLBP1*; for chloride ion transport including *BEST1,* were highly expressed in H‐DMEM (Figure 4E). However, *RPE65* and *RBP1* associated with visual cycle were highly expressed in DMEM/F12 (Figure 4E). The higher expression of *RLBP1 and BEST1* in H‐DMEM were detected at protein level; but none of difference was detected on RPE65 (Figure 4F). MERTK required for POS specific internalization was equally expressed in DMEM/F12 and H‐DMEM, but it was significantly highly expressed in H‐DMEM at protein level (Figure 4F).

Morphology, pigmentation and the expression of RPE65 protein were assessed when fhRPE‐13A cells were cultured in DMEM/F12 and H‐DMEM at day 30. Immunofluorescence of ZO‐1 showed primary fhRPE maintained mature epithelial morphology, but fhRPE‐13A, regardless of in DMEM/F12 or H‐DMEM, and ARPE‐19 lost normal epithelial morphology, developed into irregular shape and indistinct ZO‐1 staining (Figure 4G), which indicated that fhRPE‐13A and ARPE‐19 had a limited capacity to maintain polygonal epithelial morphology. And we did not find visible pigmentation with naked eyes. Importantly, RPE65 protein was detected using Western blot when fhRPE‐13A cells were cultivated in H‐DMEM after prolonged culture (Figure 4G).

We also performed the transepithelial electrical resistance (TER), a common measure of barrier function. We found that fhRPE‐13A cells, similar to ARPE‐19, could not developed a high TER and the maximum resistance was only 61 ± 5.6 Ω cm^2^ (n = 3) at day 11 (Figure 4H). The limited capacity to maintain epithelial morphology might be the reason why fhRPE‐13A and ARPE‐19 failed to reach high TER in longer time culture.

### 3.5 fhRPE‐13A cells are available for study on phagocytosis of RPE

**Figure 5:**
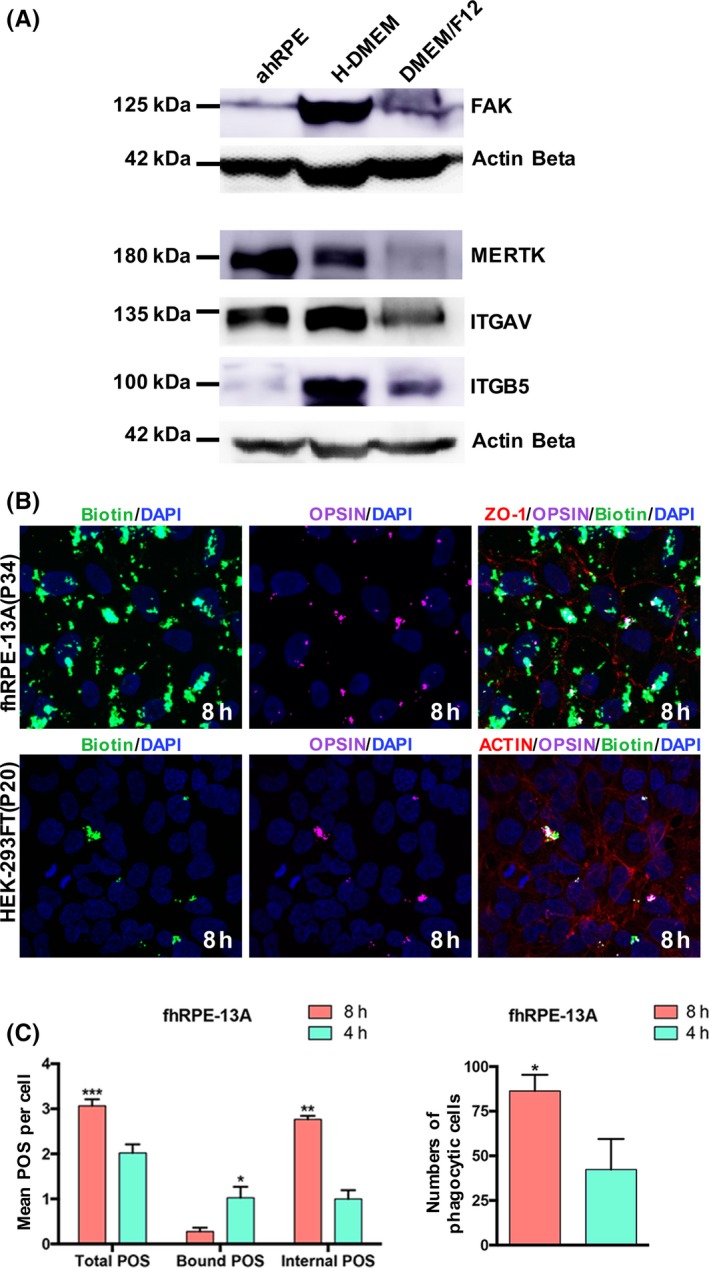
The experimental evaluation on Phagocytosis of fhRPE‐13A cells. A, Western blot analysis of proteins required for POS specific phagocytosis of fhRPE‐13A cultured in H‐DMEM or DMEM/F12, respectively. Adult RPE tissue (ahRPE) was used as control. B, Experimental evaluation on phagocytosis of fhRPE‐13A. Phagocytized porcine outer segments (only green) and surface‐bound porcine outer segments (purple and green) are shown after incubation with labeled POS for 8 h. HEK‐293FT was used as negative control. C, The number of internalized POS and phagocytic cells within 8 h *P < 0.05,**P < 0.01,***P < 0.001

Transcriptome analysis indicated that fhRPE‐13A cells might have the capacity of phagocytosis. To further confirm this hypothesis, we detected the expression of key phagocytic proteins, FAK, ITGAV, ITGB5 and MERTK, in fhRPE‐13A cells cultivated with H‐DMEM and DMEM/F12 medium using Western blot, which were implicated in the binding and internalization of outer segments. Obviously, cells in H‐DMEM highly expressed FAK, ITGAV, ITGB5 and MERTK at protein level (Figure 5A). Then, we conducted an examination of phagocytic uptake of outer segment material isolated from pig eyes to analyse the phagocytic activity of fhRPE‐13A cells in H‐DMEM at 4 hours and 8 hours, respectively. Internalized POS appeared in the green image only, and surface‐bound POS appeared in the green and purple images (Figure 5B). A large amount of internal POS was observed in fhRPE‐13A cells, while on the contrary, in HEK‐293FT cells, a negative control, none of internalized POS was detected. Furthermore, statistics suggested that incubation for 4 hours with isolated POS represented primarily bound POS; while incubation for 8 hours represented primarily internalized POS (Figure 5C). The amount of phagocytic cells within 8 hours was significantly larger than that within 4 hours (Figure 5C). Those further supported the idea that the fhRPE‐13A cells were functional RPE at phagocytosis.

### 3.6 fhRPE‐13A cells are available for study on morphogenesis of RPE

**Figure 6:**
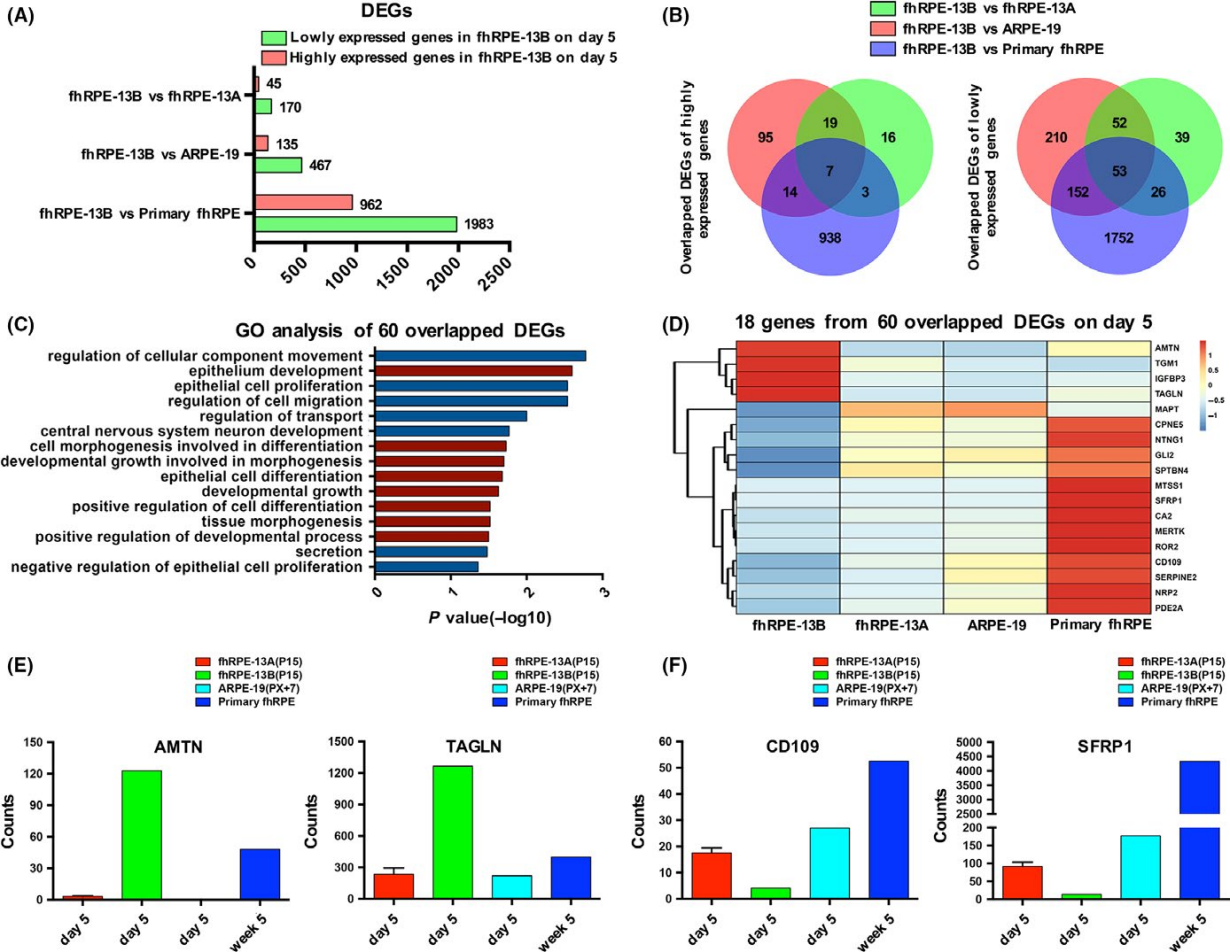
Gene expression profile analysis of DEGs by comparing fhRPE‐13B with fhRPE‐13A, ARPE‐19 and primary fhRPE, respectively. A, Bar chart shows the number of differentially expressed genes (DEGs, FC ≥ 4 or FC ≤ 0.25, FDR < 0.05, FC = Fold Change.) on day 5. B, Venn diagram shows the number of overlapped genes of highly or lowly expressed DEGs, respectively. C, The Gene Ontology (GO) analysis of all 60 overlapped DEGs. Red‐colored GO terms are associated with morphogenesis, development and differentiation. The length of bars indicates −log_10_ *P* value. D, Heatmap of the 18 genes taken out from terms associated to morphogenesis, development and differentiation. Colors represent gene expression levels: High expression (Red); Moderate expression (White); Low expression (Blue). E and F, The expression levels of mesenchymal markers (E) and negative regulators of TGFβ and Wnt signaling (F) in fhRPE‐13A, fhRPE‐13B, ARPE‐19 and primary RPE. The fhRPE‐13A, fhRPE‐13B and ARPE‐19 cells were cultured at day 5; the primary RPE cells were cultured at week 5

Immunofluorescence (Figure 1A,B and Figure S1) and GO analyses of DEGs (Figure 3D and Figure S4) verified that one of the common differences of fhRPE‐13A, ARPE‐19 or primary foetal RPE with fhRPE‐13B was their morphology. In order to explore the underlying mechanism, we determined the overlap genes using a stricter criterion of DEGs (FC ≥ 4 or FC ≤ 0.25, FDR < 0.05, FC = Fold Change) (Figure 6A), and got 60 overlapped genes (Figure 6B, Table S5) by analysing lowly and highly expressed genes, respectively. GO enrichment analysis of overlapped DEGs revealed the majority of terms were related to morphogenesis, development and differentiation (Figure 6C). A total of 18 genes were taken out from terms associated to morphogenesis (Table S6) and the expression levels were shown in heatmap (Figure 6D). Among these genes, *AMTN* (Amelotin), *TAGLN* (Transgelin), *CD109* (CD109 Molecule) and *SFRP1* (Secreted Frizzled Related Protein 1) were found to be involved in transforming growth factor beta (TGFβ) or Wnt signaling. TGFβ has been shown to stimulate EMT as well as cellular migration, thereby to induce the loss of RPE morphology.^36^ The Wnt/β‐catenin pathway is also implicated in EMT and known to participate in the loss of epithelial phenotype on several cells, including RPE.^37^ AMTN is a specifically expressed during the maturation stage of dental enamel formation,^38^ and TAGLN functions as an actin‐crosslinking/gelling protein of the calponin family.^39^ AMTN and TAGLN both are TGFβ‐inducible genes,^39^, ^40^ in fhRPE‐13B cells, their expression were sharply enhanced at day 5 implicating that the TGFβ signalling was activated. CD109, as a novel negative regulator of TGFβ signalling, plays a key role in suppression of tumourigenesis and decrease of fibrotic responses.^41^, ^42^, ^43^ SFRP1, which antagonizes the Wnt/β‐catenin pathway,^44^ its reduction results in an increased sensitivity to TGFβ signalling^45^ and therefore facilitate EMT.^46^ Based on those reports described above, the significant up–regulation of AMTN, TAGLN (Figure 6E) and down‐regulation of CD109 and SFRP1 (Figure 6F) in the fhRPE‐13B cells might explain the mechanism of morphological abnormalities.

## 4 DISCUSSION

At present, several RPE cell lines have been established and applied to research of ophthalmology.^12^, ^13^, ^14^, ^47^ It is worth mentioning that none of foetal human RPE cell lines was reported. For example, the ARPE‐19 cells derived from a man of 19‐year‐old donor,^13^ and the D407 cells come from a man of 12‐year‐old donor.^12^ In this paper, we firstly obtain two foetal human RPE cell lines, fhRPE‐13A and fhRPE‐13B, in absence of any viral transformation vectors, which express developmental factors (*MITF, PAX6* and *OTX2*) and can proliferate stably and maintain their morphology after extensive passaging.

Human RPE cells become post‐mitotic and differentiate early, approximately day 32‐50, into a pigmented monolayer epithelium, which normally remain quiescent and non‐migratory throughout life. However, proliferation was observed in vivo when RPE cells were damage or in some intraocular disease, such as proliferative vitreoretinopathy (PVR).^48^ In these conditions, RPE cells prolong to fusiform‐like morphology, called an epithelium to mesenchyme transition (EMT). The proliferation of RPE was also found in vitro, producing stable RPE progeny or performing epithelium to mesenchymal transition which produce osteogenic, chondrogenic and myogenic progeny.^7^ Interestingly, in this study, we found the fhRPE‐13A cells could re‐establish polygonal morphology, and express most RPE‐related genes, partially at a lower level than primary fhRPE but higher than ARPE‐19 cells, such as *TYR* and *TYRP1*; however, the fhRPE‐13B cells existed an atypical fusiform, contemporaneously expressed RPE genes and mesenchymal markers, meaning they were in mesenchymal state. The generation of these two novel cell lines strengthen the notion that human RPE can adopt different fates.

The range of application of cultured RPE depends on its ability to recapitulate genetic and functional characteristics of the native RPE. Via transcriptomic comparisons of fhRPE‐13A, ARPE‐19 and primary fhRPE, most phagocytosis‐associated genes were highly or equally expressed in fhRPE‐13A except *GAS6*(Growth Arrest Specific 6), which was expressed at lower level in fhRPE‐13A cells than ARPE‐19 cells, but at equal level in fhRPE‐13A cells and primary fhRPE. Furthermore, experimental results proved fhRPE‐13A cells could perform active phagocytosis. In fact, all generated RPE cell lines strikingly tend to keep phagocytic activity.^15^ Hence, we believed that fhRPE‐13A cells were normal in phagocytic activity regardless genetic or functional levels.

The cell‐cell contact initiates the epithelial phenotype development, but once a previously established monolayer is disrupted, RPE cells will lose the epithelium morphology, then proliferate and migrate to the injury regions. The research for mechanism of RPE cells re‐establishing a mature epithelial phenotype is still a challenge for biologists and ophthalmologists. To date, TGF‐β mediated EMT has been determined in RPE, which is a dominant negative determinant of RPE morphological re‐establishment and differentiation. Significantly, using receptor kinase inhibitor, A‐83‐01, to inhibit TGF‐β mediated signalling restores the mesenchymal RPE, exhibiting a substantial degree of pigmentation and epithelial morphology.^36^, ^49^ Besides, Wnt/β‐catenin pathway has been found to play a pivotal role not only during embryogenesis for tissue specification and pigmentation, but also in the mature RPE involved in morphogenesis.^37^ Activation of this pathway promotes the loss of epithelial phenotype that accompanies EMT^50^ in RPE. Recent researches proved its reduction resulted in an increased sensitivity to TGFβ signalling^45^ and thereby facilitated EMT.^46^, ^51^ In contrast, the inhibitors of the Wnt signalling pathway, such as Dickkopf 1 (DKK1)^52^significantly suppressed the proliferation and migration of RPE cells. The inhibition of EMT by secreted frizzled‐related proteins (SFRPs) have been detected in tumour cells.^46^, ^53^ Consistent with previous studies, the present results confirmed that the increased expressions of negative regulators of Wnt signalling (SFRP1) and TGFβ signalling (CD109) induced the normal phenotype of the fhRPE‐13A cells, suggesting both pathway co‐participate the regulation of re‐morphogenesis. Based on these results, the fhRPE‐13A cells are well suited for investigating the mechanism of re‐morphogenesis, despite their defect in epithelialization.

Overall, in this paper, we firstly established and characterized a foetal human RPE cell line, fhRPE‐13A, which is a meaningful complement to researching RPE phagocytosis and morphogenesis.

## AUTHOR'S CONTRIBUTION

ZS performed experiments and wrote the manuscript; ZL and SL designed the study and revised the manuscript; HW provided the samples; BG and XG analysed the data; XZ, ML, JS, CJ and ZX performed experiments; YL revised the manuscript.
