# Supplementary figures and images for: Spontaneous generation of a novel foetal human retinal pigment epithelium (RPE) cell line available for investigation on phagocytosis and morphogenesis
Source: Cell Prolif. 2017 Sep 18;50(6):e12386. doi: 10.1111/cpr.12386 (PMC6529143; doi:10.1111/cpr.12386)

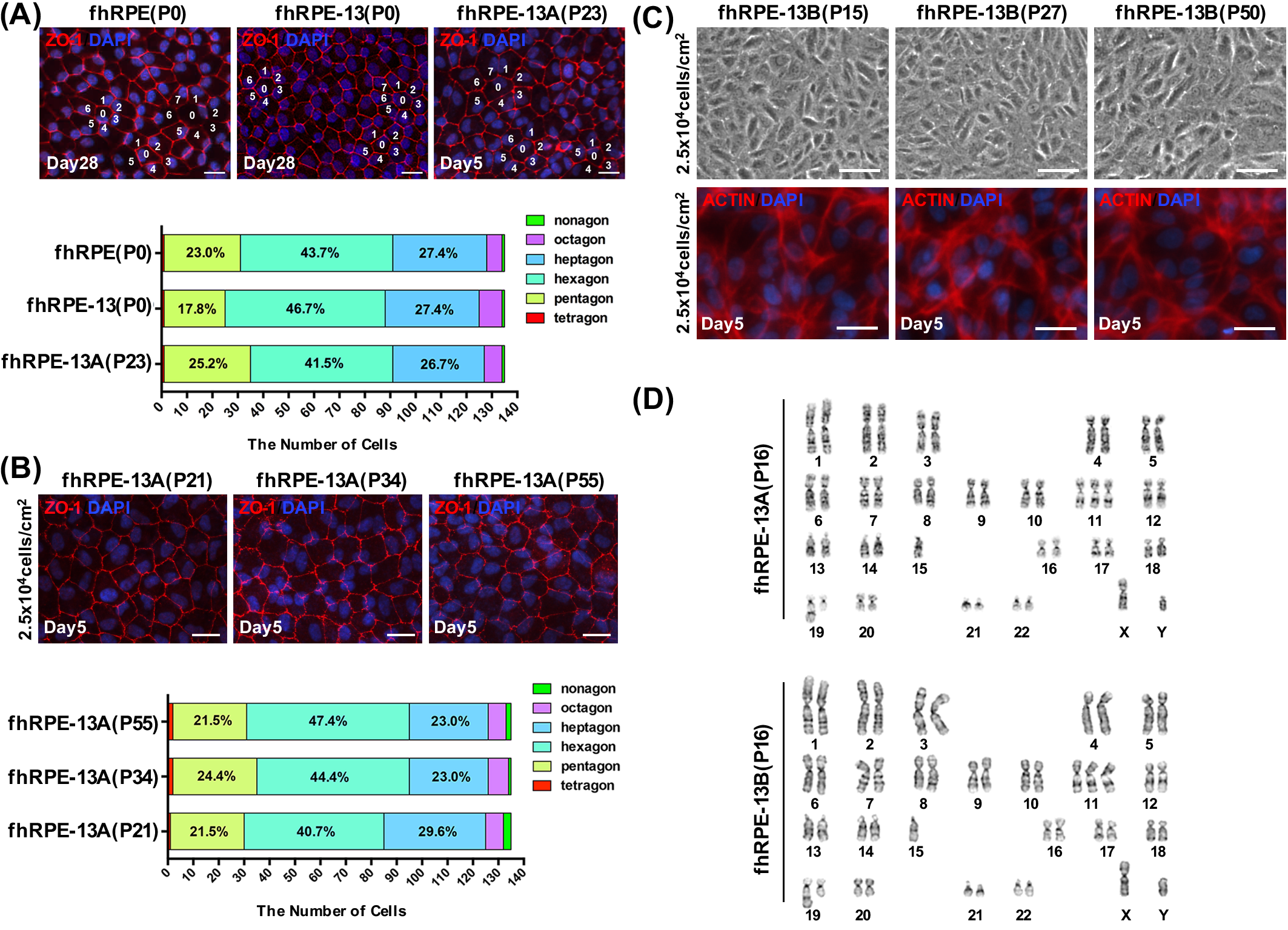

Supplement: Supplementary file 1 [file CPR-50-na-s001.tif]

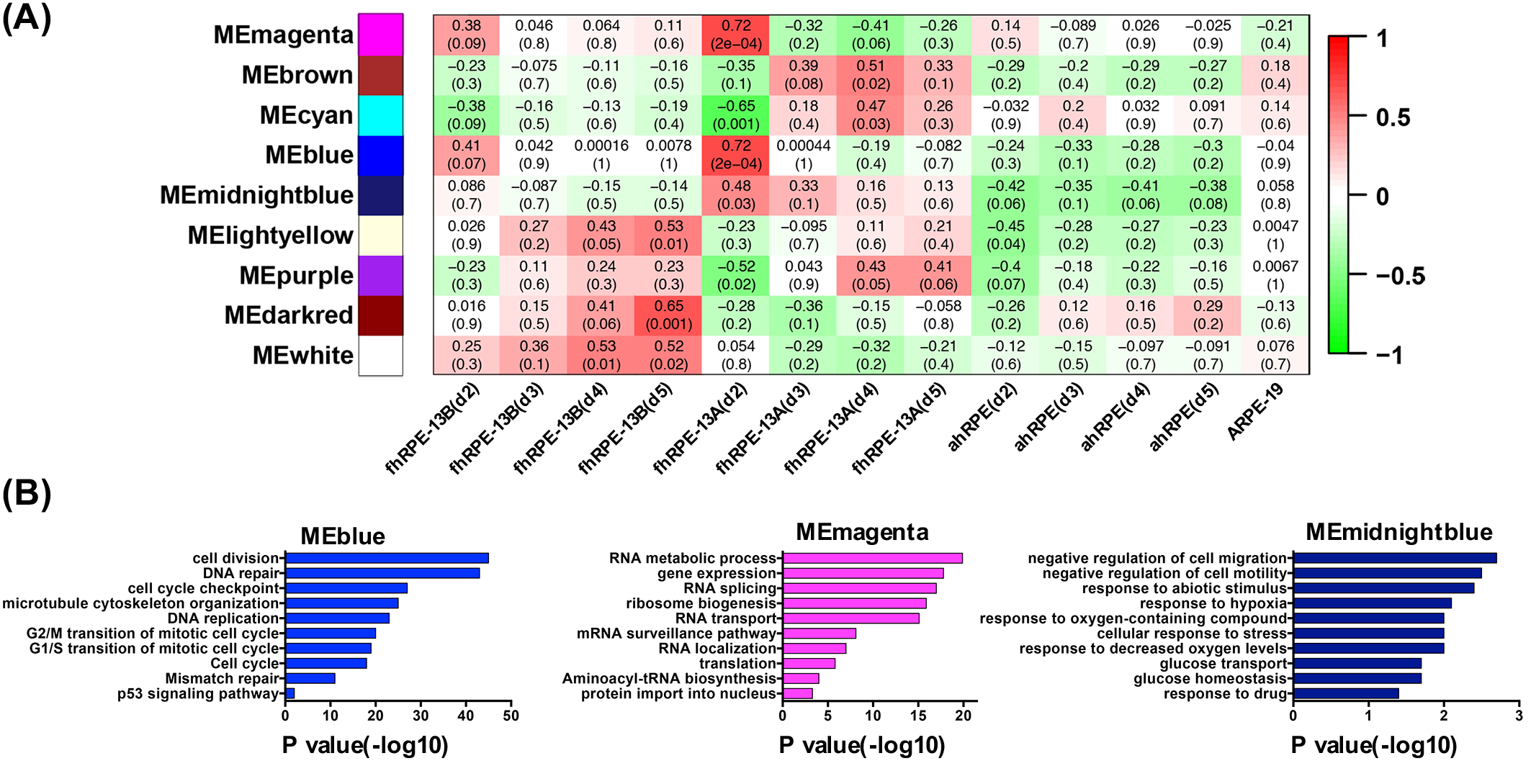

Supplement: Supplementary file 2 [file CPR-50-na-s002.tif]

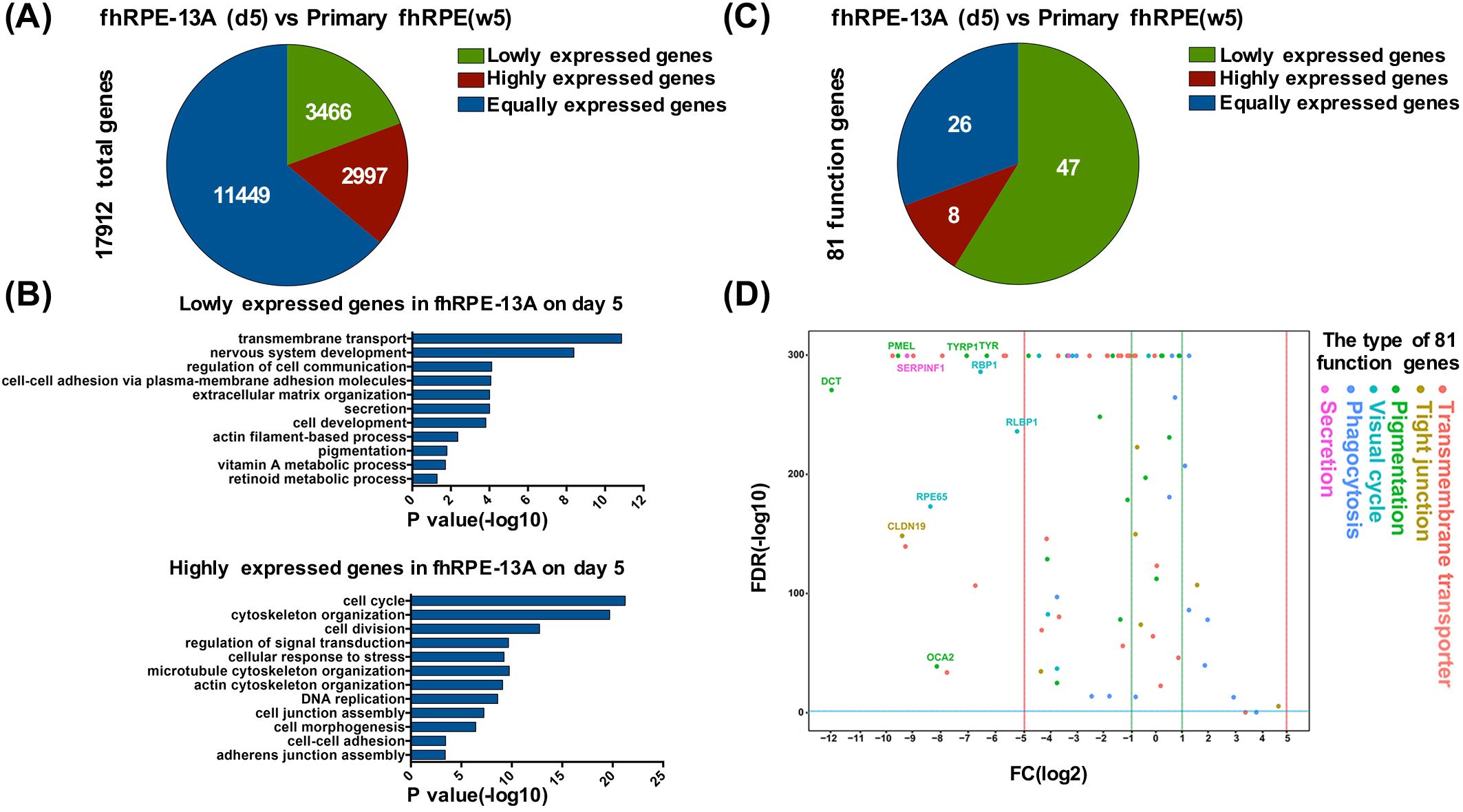

Supplement: Supplementary file 3 [file CPR-50-na-s003.tif]

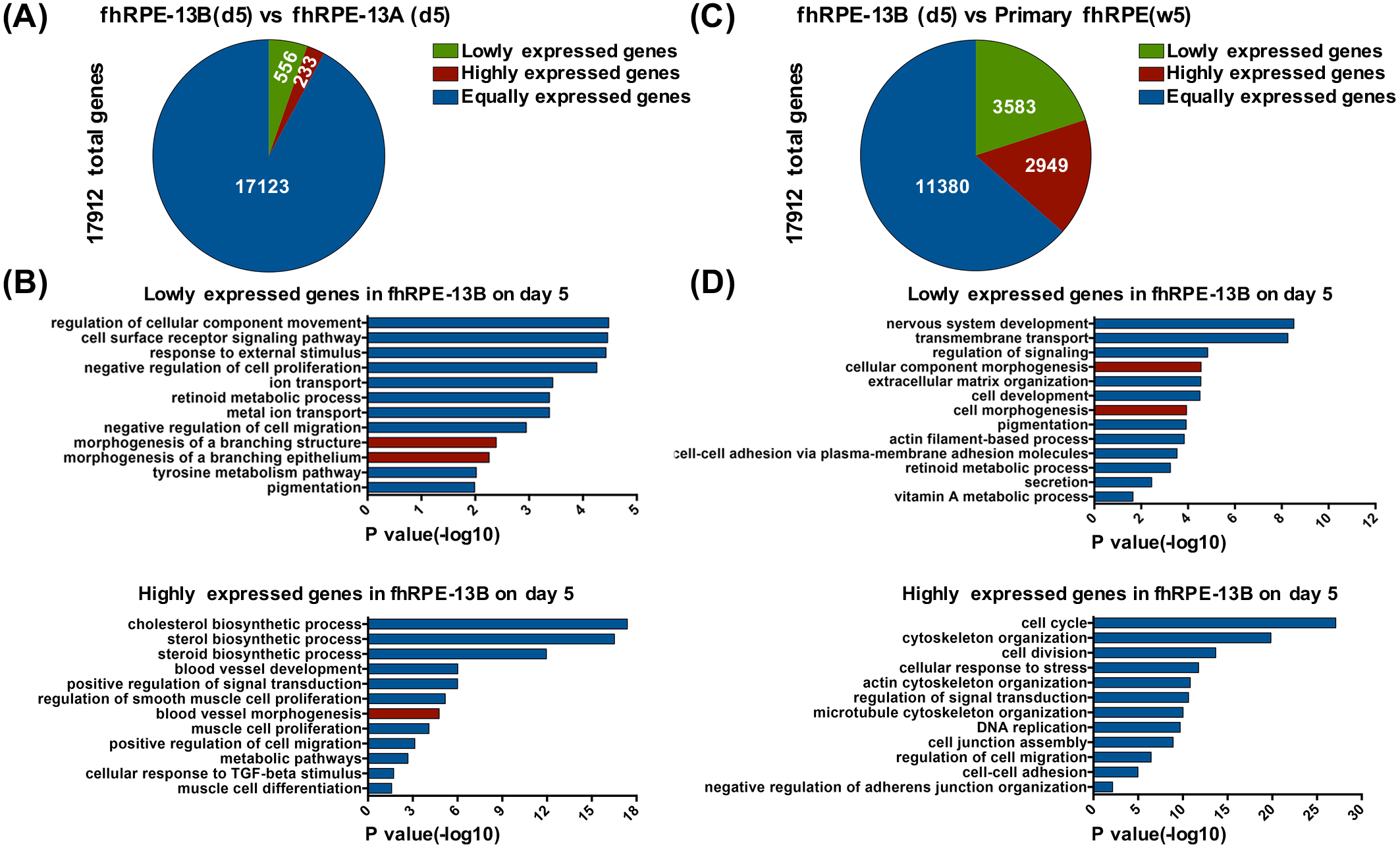

Supplement: Supplementary file 4 [file CPR-50-na-s004.tif]
