# Supplementary material for: Spontaneous generation of a novel foetal human retinal pigment epithelium (RPE) cell line available for investigation on phagocytosis and morphogenesis
Source: Cell Prolif. 2017 Sep 18;50(6):e12386. doi: 10.1111/cpr.12386 (PMC6529143; doi:10.1111/cpr.12386)
Supplement: Supplementary file 11 [file CPR-50-na-s011.docx]

**Supporting information**

References

1. Adamis AP, Shima DT, Yeo KT, et al. Synthesis and Secretion of Vascular-Permeability Factor Vascular Endothelial Growth-Factor by Human Retinal-Pigment Epithelial-Cells. *Biochem Bioph Res Co* 1993;193:631-638.

2. Adijanto J, Banzon T, Jalickee S, et al. CO2-induced ion and fluid transport in human retinal pigment epithelium. *J Gen Physiol* 2009;133:603-622.

3. Adijanto J, Castorino JJ, Wang ZX, et al. Microphthalmia-associated transcription factor (MITF) promotes differentiation of human retinal pigment epithelium (RPE) by regulating microRNAs-204/211 expression. *J Biol Chem* 2012;287:20491-20503.

4. Bakall B, Marmorstein LY, Hoppe G, et al. Expression and localization of bestrophin during normal mouse development. *Invest Ophthalmol Vis Sci* 2003;44:3622-3628.

5. Barnstable CJ, Tombran-Tink J. Neuroprotective and antiangiogenic actions of PEDF in the eye: molecular targets and therapeutic potential. *Prog Retin Eye Res* 2004;23:561-577.

6. Berson JF, Harper DC, Tenza D, et al. Pmel17 initiates premelanosome morphogenesis within multivesicular bodies. *Molecular Biology of the Cell* 2001;12:3451-3464.

7. Boissy RE, Zhao H, Oetting WS, et al. Mutation in and lack of expression of tyrosinase-related protein-1 (TRP-1) in melanocytes from an individual with brown oculocutaneous albinism: a new subtype of albinism classified as "OCA3". *Am J Hum Genet* 1996;58:1145-1156.

8. Bok D, Schibler MJ, Pushkin A, et al. Immunolocalization of electrogenic sodium-bicarbonate cotransporters pNBC1 and kNBC1 in the rat eye. *Am J Physiol Renal Physiol* 2001;281:F920-935.

9. Burgoyne T, O'Connor MN, Seabra MC, et al. Regulation of melanosome number, shape and movement in the zebrafish retinal pigment epithelium by OA1 and PMEL. *Journal of Cell Science* 2015;128:1400-1407.

10. Chang Y, Finnemann SC. Tetraspanin CD81 is required for the alpha v beta5-integrin-dependent particle-binding step of RPE phagocytosis. *J Cell Sci* 2007;120:3053-3063.

11. Chuang JZ, Chou SY, Sung CH. Chloride Intracellular Channel 4 Is Critical for the Epithelial Morphogenesis of RPE Cells and Retinal Attachment. *Molecular Biology of the Cell* 2010;21:3017-3028.

12. Damek-Poprawa M, Diemer T, Lopes VS, et al. Melanoregulin (MREG) Modulates Lysosome Function in Pigment Epithelial Cells. *Journal of Biological Chemistry* 2009;284:10877-10889.

13. De Maziere AM, Muehlethaler K, van Donselaar E, et al. The melanocytic protein Melan-A/MART-1 has a subcellular localization distinct from typical melanosomal proteins. *Traffic* 2002;3:678-693.

14. Di Pietro SM, Falcon-Perez JM, Dell'Angelica EC. Characterization of BLOC-2, a complex containing the Hermansky-Pudlak syndrome proteins HPS3, HPS5 and HPS6. *Traffic* 2004;5:276-283.

15. Du J, Miller AJ, Widlund HR, et al. MLANA/MART1 and SILV/PMEL17/GP100 are transcriptionally regulated by MITF in melanocytes and melanoma. *Am J Pathol* 2003;163:333-343.

16. Economopoulou M, Hammer J, Wang F, et al. Expression, localization, and function of junctional adhesion molecule-C (JAM-C) in human retinal pigment epithelium. *Invest Ophthalmol Vis Sci* 2009;50:1454-1463.

17. Feng L, Seymour AB, Jiang S, et al. The beta3A subunit gene (Ap3b1) of the AP-3 adaptor complex is altered in the mouse hypopigmentation mutant pearl, a model for Hermansky-Pudlak syndrome and night blindness. *Hum Mol Genet* 1999;8:323-330.

18. Feng W, Yasumura D, Matthes MT, et al. Mertk triggers uptake of photoreceptor outer segments during phagocytosis by cultured retinal pigment epithelial cells. *J Biol Chem* 2002;277:17016-17022.

19. Fernandez L, Milne RL, Pita G, et al. SLC45A2: A novel malignant melanoma-associated gene. *Hum Mutat* 2008;29:1161-1167.

20. Finnemann SC. Focal adhesion kinase signaling promotes phagocytosis of integrin-bound photoreceptors. *Embo J* 2003;22:4143-4154.

21. Folli C, Calderone V, Ottonello S, et al. Identification, retinoid binding, and x-ray analysis of a human retinol-binding protein. *Proc Natl Acad Sci U S A* 2001;98:3710-3715.

22. Futter CE, Ramalho JS, Jaissle GB, et al. The role of Rab27a in the regulation of melanosome distribution within retinal pigment epithelial cells. *Molecular Biology of the Cell* 2004;15:2264-2275.

23. Gibbs D, Azarian SM, Lillo C, et al. Role of myosin VIIa and Rab27a in the motility and localization of RPE melanosomes. *Journal of Cell Science* 2004;117:6473-6483.

24. Haeseleer F, Jang GF, Imanishi Y, et al. Dual-substrate specificity short chain retinol dehydrogenases from the vertebrate retina. *J Biol Chem* 2002;277:45537-45546.

25. Hall MO, Obin MS, Heeb M, et al. Both protein S and Gas6 stimulate outer segment phagocytosis by cultured rat retinal pigment epithelial cells. *Experimental Eye Research* 2005;81:581-591.

26. Hall MO, Prieto AL, Obin MS, et al. Outer segment phagocytosis by cultured retinal pigment epithelial cells requires Gas6. *Exp Eye Res* 2001;73:509-520.

27. Hemesath TJ, Steingrimsson E, McGill G, et al. microphthalmia, a critical factor in melanocyte development, defines a discrete transcription factor family. *Genes Dev* 1994;8:2770-2780.

28. Hoashi T, Watabe H, Muller J, et al. MART-1 is required for the function of the melanosomal matrix protein PMEL17/GP100 and the maturation of melanosomes. *Journal of Biological Chemistry* 2005;280:14006-14016.

29. Hu J, Bok D. The use of cultured human fetal retinal pigment epithelium in studies of the classical retinoid visual cycle and retinoid-based disease processes. *Exp Eye Res* 2014;126:46-50.

30. Hughes BA, Adorante JS, Miller SS, et al. Apical Electrogenic Nahco3 Cotransport - a Mechanism for Hco3 Absorption across the Retinal-Pigment Epithelium. *J Gen Physiol* 1989;94:125-150.

31. Hume AN, Collinson LM, Rapak A, et al. Rab27a regulates the peripheral distribution of melanosomes in melanocytes. *J Cell Biol* 2001;152:795-808.

32. Jin M, Yuan Q, Li S, et al. Role of LRAT on the retinoid isomerase activity and membrane association of Rpe65. *J Biol Chem* 2007;282:20915-20924.

33. Kao L, Kurtz LM, Shao X, et al. Severe neurologic impairment in mice with targeted disruption of the electrogenic sodium bicarbonate cotransporter NBCe2 (Slc4a5 gene). *J Biol Chem* 2011;286:32563-32574.

34. Kawaguchi M, Hozumi Y, Suzuki T. ADAM protease inhibitors reduce melanogenesis by regulating PMEL17 processing in human melanocytes. *J Dermatol Sci* 2015;78:133-142.

35. Kawaguchi R, Yu J, Honda J, et al. A membrane receptor for retinol binding protein mediates cellular uptake of vitamin A. *Science* 2007;315:820-825.

36. Konari K, Sawada N, Zhong Y, et al. Development of the blood-retinal barrier in vitro: formation of tight junctions as revealed by occludin and ZO-1 correlates with the barrier function of chick retinal pigment epithelial cells. *Exp Eye Res* 1995;61:99-108.

37. Kornak U, Kasper D, Bosl MR, et al. Loss of the ClC-7 chloride channel leads to osteopetrosis in mice and man. *Cell* 2001;104:205-215.

38. Kummer MP, Maruyama H, Huelsmann C, et al. Formation of Pmel17 amyloid is regulated by juxtamembrane metalloproteinase cleavage, and the resulting C-terminal fragment is a substrate for gamma-secretase. *J Biol Chem* 2009;284:2296-2306.

39. Kurihara T, Westenskow PD, Bravo S, et al. Targeted deletion of Vegfa in adult mice induces vision loss. *J Clin Invest* 2012;122:4213-4217.

40. Kushimoto T, Basrur V, Valencia J, et al. A model for melanosome biogenesis based on the purification and analysis of early melanosomes. *P Natl Acad Sci USA* 2001;98:10698-10703.

41. Lamason RL, Mohideen MAPK, Mest JR, et al. SLC24A5, a putative cation exchanger, affects pigmentation in zebrafish and humans. *Science* 2005;310:1782-1786.

42. Law AL, Ling Q, Hajjar KA, et al. Annexin A2 regulates phagocytosis of photoreceptor outer segments in the mouse retina. *Mol Biol Cell* 2009;20:3896-3904.

43. Liu XR, Ondek B, Williams DS. Mutant myosin VIIa causes defective melanosome distribution in the RPE of shaker-1 mice. *Nat Genet* 1998;19:117-118.

44. Lobato-Alvarez JA, Roldan ML, Lopez-Murillo TD, et al. The Apical Localization of Na+, K+-ATPase in Cultured Human Retinal Pigment Epithelial Cells Depends on Expression of the beta2 Subunit. *Front Physiol* 2016;7:450.

45. Luo Y, Fukuhara M, Weitzman M, et al. Expression of JAM-A, AF-6, PAR-3 and PAR-6 during the assembly and remodeling of RPE tight junctions. *Brain Res* 2006;1110:55-63.

46. Maminishkis A, Chen S, Jalickee S, et al. Confluent monolayers of cultured human fetal retinal pigment epithelium exhibit morphology and physiology of native tissue. *Invest Ophthalmol Vis Sci* 2006;47:3612-3624.

47. Mao YY, Finnemann SC. Essential diurnal Rac1 activation during retinal phagocytosis requires alpha v beta 5 integrin but not tyrosine kinases focal adhesion kinase or Mer tyrosine kinase. *Molecular Biology of the Cell* 2012;23:1104-1114.

48. Marmorstein AD, Marmorstein LY, Rayborn M, et al. Bestrophin, the product of the Best vitelliform macular dystrophy gene (VMD2), localizes to the basolateral plasma membrane of the retinal pigment epithelium. *Proc Natl Acad Sci U S A* 2000;97:12758-12763.

49. Maw MA, Kennedy B, Knight A, et al. Mutation of the gene encoding cellular retinaldehyde-binding protein in autosomal recessive retinitis pigmentosa. *Nat Genet* 1997;17:198-200.

50. Moiseyev G, Chen Y, Takahashi Y, et al. RPE65 is the isomerohydrolase in the retinoid visual cycle. *Proc Natl Acad Sci U S A* 2005;102:12413-12418.

51. Montoliu L, Marks MS. A new type of syndromic albinism associated with mutations in AP3D1. *Pigment Cell Melanoma Res* 2017;30:5-7.

52. Nandrot EF, Anand M, Almeida D, et al. Essential role for MFG-E8 as ligand for alpha v beta 5 integrin in diurnal retinal phagocytosis. *P Natl Acad Sci USA* 2007;104:12005-12010.

53. Nandrot EF, Kim Y, Brodie SE, et al. Loss of synchronized retinal phagocytosis and age-related blindness in mice lacking alphavbeta5 integrin. *J Exp Med* 2004;200:1539-1545.

54. Ooi CE, Moreira JE, Dell'Angelica EC, et al. Altered expression of a novel adaptin leads to defective pigment granule biogenesis in the Drosophila eye color mutant garnet. *Embo J* 1997;16:4508-4518.

55. Palmisano I, Bagnato P, Palmigiano A, et al. The ocular albinism type 1 protein, an intracellular G protein-coupled receptor, regulates melanosome transport in pigment cells. *Hum Mol Genet* 2008;17:3487-3501.

56. Parker MD, Boron WF. The divergence, actions, roles, and relatives of sodium-coupled bicarbonate transporters. *Physiol Rev* 2013;93:803-959.

57. Peng S, Rao VS, Adelman RA, et al. Claudin-19 and the barrier properties of the human retinal pigment epithelium. *Invest Ophthalmol Vis Sci* 2011;52:1392-1403.

58. Philp NJ, Wang D, Yoon H, et al. Polarized expression of monocarboxylate transporters in human retinal pigment epithelium and ARPE-19 cells. *Invest Ophthalmol Vis Sci* 2003;44:1716-1721.

59. Philp NJ, Yoon H, Grollman EF. Monocarboxylate transporter MCT1 is located in the apical membrane and MCT3 in the basal membrane of rat RPE. *Am J Physiol-Reg I* 1998;274:R1824-R1828.

60. Rachel RA, Mason CA, Beermann F. Influence of tyrosinase levels on pigment accumulation in the retinal pigment epithelium and on the uncrossed retinal projection. *Pigment Cell Res* 2002;15:273-281.

61. Rakoczy PE, Lai CM, Baines M, et al. Modulation of cathepsin D activity in retinal pigment epithelial cells. *Biochem J* 1997;324 ( Pt 3):935-940.

62. Rakoczy PE, Lai MC, Baines MG, et al. Expression of cathepsin S antisense transcripts by adenovirus in retinal pigment epithelial cells. *Invest Ophthalmol Vis Sci* 1998;39:2095-2104.

63. Rinchik EM, Bultman SJ, Horsthemke B, et al. A Gene for the Mouse Pink-Eyed Dilution Locus and for Human Type-Ii Oculocutaneous Albinism. *Nature* 1993;361:72-76.

64. Rochin L, Hurbain I, Serneels L, et al. BACE2 processes PMEL to form the melanosome amyloid matrix in pigment cells. *Proc Natl Acad Sci U S A* 2013;110:10658-10663.

65. Ruiz A, Bhat SP, Bok D. Characterization and quantification of full-length and truncated Na,K-ATPase alpha 1 and beta 1 RNA transcripts expressed in human retinal pigment epithelium. *Gene* 1995;155:179-184.

66. Ruiz A, Bhat SP, Bok D. Expression and synthesis of the Na,K-ATPase beta 2 subunit in human retinal pigment epithelium. *Gene* 1996;176:237-242.

67. Ruiz A, Winston A, Lim YH, et al. Molecular and biochemical characterization of lecithin retinol acyltransferase. *J Biol Chem* 1999;274:3834-3841.

68. Ryeom SW, Sparrow JR, Silverstein RL. CD36 participates in the phagocytosis of rod outer segments by retinal pigment epithelium. *Journal of Cell Science* 1996;109:387-395.

69. Setty SR, Tenza D, Truschel ST, et al. BLOC-1 is required for cargo-specific sorting from vacuolar early endosomes toward lysosome-related organelles. *Mol Biol Cell* 2007;18:768-780.

70. Simon A, Romert A, Gustafson AL, et al. Intracellular localization and membrane topology of 11-cis retinol dehydrogenase in the retinal pigment epithelium suggest a compartmentalized synthesis of 11-cis retinaldehyde. *J Cell Sci* 1999;112 ( Pt 4):549-558.

71. Sitaram A, Piccirillo R, Palmisano I, et al. Localization to Mature Melanosomes by Virtue of Cytoplasmic Dileucine Motifs Is Required for Human OCA2 Function. *Molecular Biology of the Cell* 2009;20:1464-1477.

72. Stamer WD, Bok D, Hu J, et al. Aquaporin-1 channels in human retinal pigment epithelium: Role in transepithelial water movement. *Invest Ophth Vis Sci* 2003;44:2803-2808.

73. Strick DJ, Feng W, Vollrath D. Mertk Drives Myosin II Redistribution during Retinal Pigment Epithelial Phagocytosis. *Invest Ophth Vis Sci* 2009;50:2427-2435.

74. Strunnikova NV, Maminishkis A, Barb JJ, et al. Transcriptome analysis and molecular signature of human retinal pigment epithelium. *Hum Mol Genet* 2010;19:2468-2486.

75. Sugasawa K, Deguchi J, Okami T, et al. Immunocytochemical analyses of distributions of Na, K-ATPase and GLUT1, insulin and transferrin receptors in the developing retinal pigment epithelial cells. *Cell Struct Funct* 1994;19:21-28.

76. Sun M, Finnemann SC, Febbraio M, et al. Light-induced oxidation of photoreceptor outer segment phospholipids generates ligands for CD36-mediated phagocytosis by retinal pigment epithelium: a potential mechanism for modulating outer segment phagocytosis under oxidant stress conditions. *J Biol Chem* 2006;281:4222-4230.

77. Theos AC, Tenza D, Martina JA, et al. Functions of adaptor protein (AP)-3 and AP-1 in tyrosinase sorting from endosomes to melanosomes. *Mol Biol Cell* 2005;16:5356-5372.

78. Tombran-Tink J, Shivaram SM, Chader GJ, et al. Expression, secretion, and age-related downregulation of pigment epithelium-derived factor, a serpin with neurotrophic activity. *J Neurosci* 1995;15:4992-5003.

79. Tran TL, Bek T, Holm L, et al. Aquaporins 6-12 in the human eye. *Acta Ophthalmol* 2013;91:557-563.

80. van Niel G, Bergam P, Di Cicco A, et al. Apolipoprotein E Regulates Amyloid Formation within Endosomes of Pigment Cells. *Cell Rep* 2015;13:43-51.

81. van Niel G, Charrin S, Simoes S, et al. The tetraspanin CD63 regulates ESCRT-independent and -dependent endosomal sorting during melanogenesis. *Dev Cell* 2011;21:708-721.

82. Weng TX, Godley BF, Jin GF, et al. Oxidant and antioxidant modulation of chloride channels expressed in human retinal pigment epithelium. *Am J Physiol Cell Physiol* 2002;283:C839-849.

83. Westenskow PD, Moreno SK, Krohne TU, et al. Using Flow Cytometry to Compare the Dynamics of Photoreceptor Outer Segment Phagocytosis in iPS-Derived RPE Cells. *Invest Ophth Vis Sci* 2012;53:6282-6290.

84. Wills NK, Weng T, Mo L, et al. Chloride channel expression in cultured human fetal RPE cells: response to oxidative stress. *Invest Ophthalmol Vis Sci* 2000;41:4247-4255.

85. Wimmers S, Strauss O. Basal calcium entry in retinal pigment epithelial cells is mediated by TRPC channels. *Invest Ophth Vis Sci* 2007;48:5767-5772.

86. Wu BX, Chen Y, Chen Y, et al. Cloning and characterization of a novel all-trans retinol short-chain dehydrogenase/reductase from the RPE. *Invest Ophthalmol Vis Sci* 2002;43:3365-3372.

87. Yang DL, Swarninathan A, Zhang XM, et al. Expression of Kir7.1 and a novel Kir7.1 splice variant in native human retinal pigment epithelium. *Experimental Eye Research* 2008;86:81-91.

88. Zhang X, Yang D, Hughes BA. KCNQ5/K(v)7.5 potassium channel expression and subcellular localization in primate retinal pigment epithelium and neural retina. *Am J Physiol Cell Physiol* 2011;301:C1017-1026.

89. Zhao PY, Gan G, Peng S, et al. TRP Channels Localize to Subdomains of the Apical Plasma Membrane in Human Fetal Retinal Pigment Epithelium. *Invest Ophthalmol Vis Sci* 2015;56:1916-1923.
